# Distrontium trimanganese(II) bis­(hydro­gen­phosphate) bis­(ortho­phosphate)

**DOI:** 10.1107/S1600536813018898

**Published:** 2013-07-13

**Authors:** Jamal Khmiyas, Abderrazzak Assani, Mohamed Saadi, Lahcen El Ammari

**Affiliations:** a Laboratoire de Chimie du Solide Appliquée, Faculté des Sciences, Université Mohammed V-Agdal, Avenue Ibn Battouta, BP 1014, Rabat, Morocco

## Abstract

The title compound, Sr_2_Mn_3_(HPO_4_)_2_(PO_4_)_2_, was synthesized under hydro­thermal conditions. In the structure, one of two Mn atoms is located on an inversion centre, whereas all others atoms are located in general positions. The framework structure is built up from two types of MnO_6_ octa­hedra (one almost undistorted, one considerably distorted), one PO_3_OH and one PO_4_ tetra­hedron. The centrosymmetric MnO_6_ octa­hedron is linked to two other MnO_6_ octa­hedra by edge-sharing, forming infinite zigzag chains parallel to [010]. The PO_3_OH and PO_4_ tetra­hedra connect these chains through common vertices or edges, resulting in the formation of sheets parallel to (100). The Sr^2+^ cation is located in the inter­layer space and is bonded to nine O atoms in form of a distorted polyhedron and enhances the cohesion of the layers. Additional stabilization is achieved by a strong inter­layer O—H⋯O hydrogen bond between the PO_3_OH and PO_4_ units. The structure of the title phosphate is isotypic to that of Pb_2_Mn_3_(HPO_4_)_2_(PO_4_)_2_.

## Related literature

For isotypic Pb_2_Mn_3_(HPO_4_)_2_(PO_4_)_2_, see: Assani *et al.* (2012*b* ▶). For related structures, see: Assani *et al.* (2012*a* ▶); Effenberger (1999 ▶). For the thermal stability of similar compounds, see: Morozov *et al.* (2003 ▶). For applications of phosphates, see: Cheetham *et al.* (1999 ▶); Viter & Nagornyi (2009 ▶); Forster *et al.* (2003 ▶); Clearfield (1988 ▶); Joschi *et al.* (2008 ▶); Trad *et al.* (2010 ▶).

## Experimental

### 

#### Crystal data

Sr_2_Mn_3_(HPO_4_)_2_(PO_4_)_2_
*M* *_r_* = 721.96
Monoclinic, 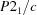

*a* = 7.8535 (1) Å
*b* = 8.7793 (2) Å
*c* = 9.6165 (2) Å
β = 101.434 (1)°
*V* = 649.88 (2) Å^3^
*Z* = 2
Mo *K*α radiation
μ = 11.58 mm^−1^
*T* = 296 K
0.33 × 0.24 × 0.12 mm

#### Data collection

Bruker APEXII CCD diffractometer
Absorption correction: multi-scan (*SADABS*; Bruker, 2009 ▶) *T*_min_ = 0.046, *T*_max_ = 0.215
12425 measured reflections
3138 independent reflections
2874 reflections with *I* > 2σ(*I*)
*R*_int_ = 0.025

#### Refinement

*R*[*F* ^2^ > 2σ(*F* ^2^)] = 0.019
*wR*(*F* ^2^) = 0.045
*S* = 1.06
3138 reflections
115 parameters
H-atom parameters constrained
Δρ_max_ = 0.58 e Å^−3^
Δρ_min_ = −0.54 e Å^−3^

### 

Data collection: *APEX2* (Bruker, 2009 ▶); cell refinement: *SAINT* (Bruker, 2009 ▶); data reduction: *SAINT*; program(s) used to solve structure: *SHELXS97* (Sheldrick, 2008 ▶); program(s) used to refine structure: *SHELXL97* (Sheldrick, 2008 ▶); molecular graphics: *ORTEP-3 for Windows* (Farrugia, 2012 ▶) and *DIAMOND* (Brandenburg, 2006 ▶); software used to prepare material for publication: *publCIF* (Westrip, 2010 ▶).

## Supplementary Material

Crystal structure: contains datablock(s) I, global. DOI: 10.1107/S1600536813018898/wm2758sup1.cif

Structure factors: contains datablock(s) I. DOI: 10.1107/S1600536813018898/wm2758Isup2.hkl

Additional supplementary materials:  crystallographic information; 3D view; checkCIF report

## Figures and Tables

**Table 1:** Hydrogen-bond geometry (Å, °)

*D*—H⋯*A*	*D*—H	H⋯*A*	*D*⋯*A*	*D*—H⋯*A*
O8—H8⋯O4	0.82	1.66	2.4828 (14)	177

**Fig. 1.**
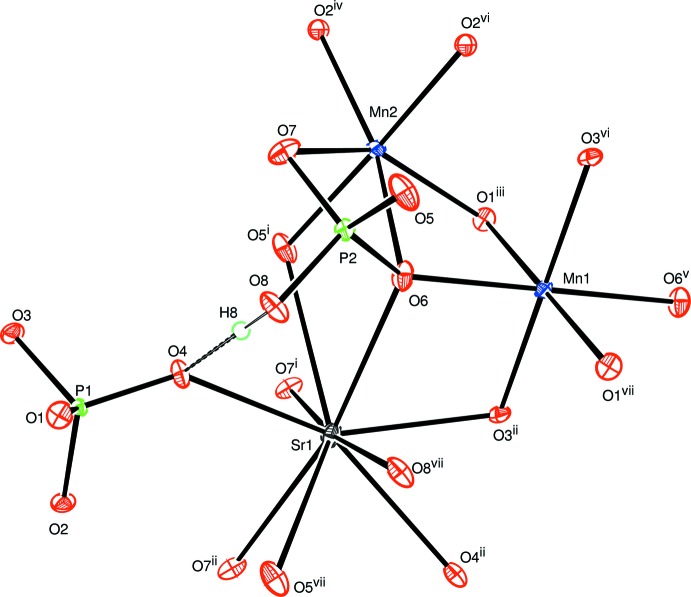
: A partial plot of the crystal structure of the title compound. Displacement ellipsoids are drawn at the 50% probability level. Symmetry codes: (i) -x + 1, y - 1/2, -z + 1/2; (ii) -x + 1, -y + 1, -z + 1; (iii) x, -y + 3/2, z - 1/2; (iv) -x + 2, -y + 1, -z + 1; (v) x + 1, -y + 3/2, z + 1/2; (vi) x + 1, y, z; (vii) -x + 1, y + 1/2, -z + 1/2; (viii) x, -y + 3/2, z + 1/2.

**Fig. 2.**
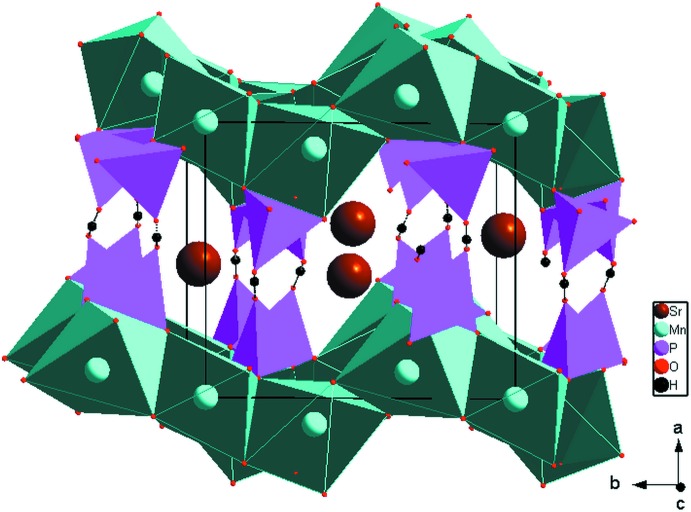
: Polyhedral representation of Sr2Mn3(HPO4)2(PO4)2, showing Sr2+ cations between layers and O—H···O hydrogen bonds (dashed lines) between the sheets.

## supplementary crystallographic information

### Comment

Widespread studies were devoted to metal-based phosphates, either with open-framework structures, or in terms of porous materials. Within those materials, the anionic framework, generally constructed from PO_4_ tetrahedra connected to metal (*M*) cations in different coordination environments *M*O*_n_* (with *n* = 4, 5 and 6), can generate pores and channels offering a suitable environment to accommodate various other cations. Besides their high chemical activity and their thermal stability (Morozov *et al.*, 2003), such metal-based phosphates have some interesting properties leading to applications such as in catalysis (Cheetham *et al.*, 1999; Viter & Nagornyi, 2009), ion-exchangers (Clearfield, 1988; Joschi *et al.*, 2008), gas sorption (Forster *et al.*, 2003), or batteries (Trad *et al.*, 2010).

Our interest is particularly focused on hydrothermally synthesized orthophosphates within the ternary systems *M*O–*M*'O–P_2_O_5_ with *M* and *M*' = divalent cations. We have recently characterized some new lead cobalt or manganese phosphates, *viz*. Co_2_Pb(HPO_4_)(PO_4_)OH^.^H_2_O (Assani *et al.*, 2012*a*) and Pb_2_Mn_3_(HPO_4_)_2_(PO_4_)_2_ (Assani *et al.*, 2012*b*). In line with the focus of our research, the present paper describes the hydrothermal synthesis and the structural characterization of a new strontium manganese phosphate, Sr_2_Mn_3_(HPO_4_)_2_(PO_4_)_2_, that is isotypic with its lead analogue, Pb_2_Mn_3_(HPO_4_)_2_(PO_4_)_2_. These two phosphates are characterized by an Mn:P ratio = 3:4, which is rarely observed, with the exception of some copper-based orthophosphates, Pb_3_Cu_3_(PO_4_)_4_ and Sr_3_Cu_3_(PO_4_)_4_ (Effenberger, 1999), also with Cu:P = 3:4.

In the structure of the title compound, one of the two manganese sites (Mn1) is located on a centre of inversion, while all remaining atoms are in general positions. A part of the structure, as given in Fig. 1, shows the different types of polyhedra around the metal positions and the P atoms. The centrosymmetric Mn1O_6_ octahedron is linked to two distorted Mn2O_6_ octahedra by a common edge, thus forming infinite zigzag chains with composition [Mn_3_O_14_]_∞_ running parallel to [010] (Fig. 2). Adjacent chains are linked to each other through PO_4_ and PO_3_OH tetrahedra, *via* common corners or edges, leading to the formation of layers parallel to (100). The cohesion of the crystal structure is ensured on one hand by the presence of the Sr^2+^ cations in the interlayer space and on the other hand by strong O—H···O hydrogen bonds between sheets (Fig. 2 and Table 2).

In the structure of the title compound, the Sr^2+^ cation is surrounded by nine O atoms instead of eight as in the case of Pb_2_Mn_3_(HPO_4_)_2_(PO_4_)_2_. All other bond lengths and angles are similar in the two structures, with the exception of the Mn2—O bond lengths. In the title structure, four medium-long bonds in the range 2.1189 (10) to 2.1875 (9) Å and two longer bonds of 2.4079 (12) and 2.4609 (11) Å are observed, whereas in the lead analogue five medium-long bonds in the range 2.094 (4) to 2.235 (4) Å and one considerably long bond of 2.610 (4) Å is observed.

### Experimental

Transparent crystals of Sr_2_Mn_3_(HPO_4_)_2_(PO_4_)_2_ were isolated from hydrothermal treatment of the reaction mixture of strontium, manganese, sodium and phosphate precursors in a proportion corresponding to the molar ratio Sr: Mn: Na: P: = 4: 4.5: 1: 6. The hydrothermal reaction was conducted in a 23 ml Teflon-lined autoclave filled to 50% with distilled water and under autogenously pressure at 473 K for five days. After being filtered off, washed with deionized water and air-dried, the reaction product consisted of colourless crystals with a platy form.

### Refinement

The O-bound H atom was initially located in a difference map and refined with O—H distance restraint of 0.82 (1) Å. In the last cycle it was refined in the riding model approximation with *U*_iso_(H) set to 1.5Ueq(O). The highest peak and the deepest hole in the final Fourier map are at 0.64 Å and 0.55 Å, from Mn2.

### Crystal data

**Table:**

Sr_2_Mn_3_(HPO_4_)_2_(PO_4_)_2_	*F*(000) = 682
*M**_r_* = 721.96	*D*_x_ = 3.689 Mg m^−^^3^
Monoclinic, *P*2_1_/*c*	Mo *K*α radiation, λ = 0.71073 Å
Hall symbol: -P 2ybc	Cell parameters from 3138 reflections
*a* = 7.8535 (1) Å	θ = 2.7–36.3°
*b* = 8.7793 (2) Å	µ = 11.58 mm^−^^1^
*c* = 9.6165 (2) Å	*T* = 296 K
β = 101.434 (1)°	Sheet, colourless
*V* = 649.88 (2) Å^3^	0.33 × 0.24 × 0.12 mm
*Z* = 2	

### Data collection

**Table:**

Bruker APEXII CCD diffractometer	3138 independent reflections
Radiation source: fine-focus sealed tube	2874 reflections with *I* > 2σ(*I*)
Graphite monochromator	*R*_int_ = 0.025
φ and ω scans	θ_max_ = 36.3°, θ_min_ = 2.7°
Absorption correction: multi-scan (*SADABS*; Bruker (2009)	*h* = −13→12
*T*_min_ = 0.046, *T*_max_ = 0.215	*k* = −14→14
12425 measured reflections	*l* = −16→16

### Refinement

**Table:**

Refinement on *F*^2^	Primary atom site location: structure-invariant direct methods
Least-squares matrix: full	Secondary atom site location: difference Fourier map
*R*[*F*^2^ > 2σ(*F*^2^)] = 0.019	Hydrogen site location: difference Fourier map
*wR*(*F*^2^) = 0.045	H-atom parameters constrained
*S* = 1.06	*w* = 1/[σ^2^(*F*_o_^2^) + (0.0211*P*)^2^ + 0.239*P*] where *P* = (*F*_o_^2^ + 2*F*_c_^2^)/3
3138 reflections	(Δ/σ)_max_ = 0.002
115 parameters	Δρ_max_ = 0.58 e Å^−^^3^
0 restraints	Δρ_min_ = −0.54 e Å^−^^3^

### Special details

**Table:**

Geometry. All s.u.'s (except the s.u. in the dihedral angle between two l.s. planes) are estimated using the full covariance matrix. The cell s.u.'s are taken into account individually in the estimation of s.u.'s in distances, angles and torsion angles; correlations between s.u.'s in cell parameters are only used when they are defined by crystal symmetry. An approximate (isotropic) treatment of cell s.u.'s is used for estimating s.u.'s involving l.s. planes.
Refinement. Refinement of *F*^2^ against all reflections. The weighted *R*-factor *wR* and goodness of fit *S* are based on *F*^2^, conventional *R*-factors *R* are based on *F*, with *F* set to zero for negative *F*^2^. The threshold expression of *F*^2^ > 2σ(*F*^2^) is used only for calculating *R*-factors(gt) *etc*. and is not relevant to the choice of reflections for refinement. *R*-factors based on *F*^2^ are statistically about twice as large as those based on *F*, and *R*- factors based on all data will be even larger.

### Fractional atomic coordinates and isotropic or equivalent isotropic displacement parameters (Å^2^)

**Table:**

	*x*	*y*	*z*	*U*_iso_*/*U*_eq_	
Sr1	0.573794 (17)	0.478038 (15)	0.234323 (14)	0.00999 (3)	
Mn1	1.0000	0.5000	0.5000	0.00677 (5)	
Mn2	0.89650 (3)	0.85638 (2)	0.40357 (2)	0.00891 (4)	
P1	0.14462 (4)	0.70256 (4)	0.22974 (3)	0.00542 (6)	
P2	0.65079 (4)	0.71451 (4)	0.56233 (3)	0.00649 (6)	
O1	0.06059 (13)	0.67784 (12)	0.35944 (10)	0.00966 (17)	
O2	0.06622 (13)	0.59473 (11)	0.10796 (10)	0.01019 (17)	
O3	0.11920 (13)	0.86980 (11)	0.18530 (11)	0.01016 (17)	
O4	0.34252 (13)	0.67330 (12)	0.27411 (10)	0.01050 (17)	
O5	0.70244 (14)	0.69325 (14)	0.72158 (10)	0.0141 (2)	
O6	0.75819 (13)	0.62546 (12)	0.47491 (11)	0.01179 (18)	
O7	0.65059 (15)	0.88211 (12)	0.51673 (11)	0.01383 (19)	
O8	0.45824 (13)	0.65486 (13)	0.53325 (11)	0.01209 (19)	
H8	0.4169	0.6607	0.4482	0.018*	

### Atomic displacement parameters (Å^2^)

**Table:**

	*U*^11^	*U*^22^	*U*^33^	*U*^12^	*U*^13^	*U*^23^
Sr1	0.00960 (6)	0.01026 (6)	0.01085 (5)	0.00204 (4)	0.00384 (4)	0.00216 (4)
Mn1	0.00742 (11)	0.00597 (11)	0.00692 (11)	0.00076 (8)	0.00144 (8)	−0.00002 (8)
Mn2	0.00962 (9)	0.00758 (9)	0.00837 (8)	0.00006 (6)	−0.00103 (6)	−0.00030 (6)
P1	0.00572 (12)	0.00557 (12)	0.00475 (12)	−0.00013 (10)	0.00052 (9)	0.00000 (9)
P2	0.00632 (13)	0.00796 (13)	0.00540 (12)	0.00034 (10)	0.00167 (10)	−0.00007 (10)
O1	0.0108 (4)	0.0108 (4)	0.0085 (4)	0.0003 (3)	0.0047 (3)	0.0012 (3)
O2	0.0137 (4)	0.0079 (4)	0.0073 (4)	−0.0001 (3)	−0.0019 (3)	−0.0020 (3)
O3	0.0126 (4)	0.0057 (4)	0.0109 (4)	0.0000 (3)	−0.0007 (3)	0.0021 (3)
O4	0.0064 (4)	0.0154 (5)	0.0093 (4)	0.0019 (3)	0.0007 (3)	0.0004 (3)
O5	0.0105 (4)	0.0256 (6)	0.0055 (4)	−0.0009 (4)	0.0002 (3)	0.0008 (4)
O6	0.0109 (4)	0.0151 (5)	0.0104 (4)	0.0054 (4)	0.0047 (3)	−0.0002 (3)
O7	0.0202 (5)	0.0076 (4)	0.0148 (4)	−0.0001 (4)	0.0061 (4)	0.0003 (3)
O8	0.0074 (4)	0.0203 (5)	0.0083 (4)	−0.0033 (4)	0.0008 (3)	0.0009 (3)

### Geometric parameters (Å, º)

**Table:**

Sr1—O3^i^	2.5641 (10)	Mn2—O1^vi^	2.1248 (10)
Sr1—O4	2.5806 (10)	Mn2—O5^iii^	2.1256 (10)
Sr1—O8^ii^	2.5775 (11)	Mn2—O2^v^	2.1875 (9)
Sr1—O7^iii^	2.5981 (11)	Mn2—O7	2.4079 (12)
Sr1—O5^ii^	2.7409 (11)	Mn2—O6	2.4609 (11)
Sr1—O4^i^	2.7599 (11)	P1—O3	1.5312 (10)
Sr1—O6	2.7923 (10)	P1—O2	1.5365 (10)
Sr1—O7^i^	2.8207 (11)	P1—O1	1.5377 (10)
Sr1—O5^iii^	3.0680 (12)	P1—O4	1.5494 (10)
Mn1—O6	2.1670 (10)	P2—O5	1.5160 (10)
Mn1—O6^iv^	2.1670 (10)	P2—O6	1.5201 (11)
Mn1—O3^v^	2.1672 (9)	P2—O7	1.5352 (11)
Mn1—O3^i^	2.1672 (9)	P2—O8	1.5721 (11)
Mn1—O1^ii^	2.1786 (10)	P2—Sr1^viii^	3.2838 (4)
Mn1—O1^vi^	2.1786 (10)	O8—H8	0.8200
Mn2—O2^vii^	2.1189 (10)		
			
O3^i^—Sr1—O4	148.09 (3)	O6^iv^—Mn1—O3^i^	92.85 (4)
O3^i^—Sr1—O8^ii^	79.54 (3)	O3^v^—Mn1—O3^i^	180.0
O4—Sr1—O8^ii^	88.83 (3)	O6—Mn1—O1^ii^	97.99 (4)
O3^i^—Sr1—O7^iii^	93.63 (3)	O6^iv^—Mn1—O1^ii^	82.01 (4)
O4—Sr1—O7^iii^	95.05 (3)	O3^v^—Mn1—O1^ii^	88.84 (4)
O8^ii^—Sr1—O7^iii^	172.09 (3)	O3^i^—Mn1—O1^ii^	91.16 (4)
O3^i^—Sr1—O5^ii^	118.41 (3)	O6—Mn1—O1^vi^	82.01 (4)
O4—Sr1—O5^ii^	74.91 (4)	O6^iv^—Mn1—O1^vi^	97.99 (4)
O8^ii^—Sr1—O5^ii^	53.21 (3)	O3^v^—Mn1—O1^vi^	91.16 (4)
O7^iii^—Sr1—O5^ii^	134.52 (3)	O3^i^—Mn1—O1^vi^	88.84 (4)
O3^i^—Sr1—O4^i^	55.56 (3)	O1^ii^—Mn1—O1^vi^	180.0
O4—Sr1—O4^i^	145.79 (2)	O2^vii^—Mn2—O1^vi^	128.50 (4)
O8^ii^—Sr1—O4^i^	69.60 (3)	O2^vii^—Mn2—O5^iii^	104.08 (4)
O7^iii^—Sr1—O4^i^	109.86 (3)	O1^vi^—Mn2—O5^iii^	92.78 (4)
O5^ii^—Sr1—O4^i^	70.92 (3)	O2^vii^—Mn2—O2^v^	77.72 (4)
O3^i^—Sr1—O6	67.65 (3)	O1^vi^—Mn2—O2^v^	92.21 (4)
O4—Sr1—O6	80.44 (3)	O5^iii^—Mn2—O2^v^	171.78 (4)
O8^ii^—Sr1—O6	67.36 (3)	O2^vii^—Mn2—O7	93.58 (4)
O7^iii^—Sr1—O6	106.45 (3)	O1^vi^—Mn2—O7	137.06 (4)
O5^ii^—Sr1—O6	114.99 (3)	O5^iii^—Mn2—O7	83.28 (4)
O4^i^—Sr1—O6	112.72 (3)	O2^v^—Mn2—O7	88.62 (4)
O3^i^—Sr1—O7^i^	122.54 (3)	O2^vii^—Mn2—O6	154.34 (4)
O4—Sr1—O7^i^	89.23 (3)	O1^vi^—Mn2—O6	76.51 (4)
O8^ii^—Sr1—O7^i^	117.08 (3)	O5^iii^—Mn2—O6	77.09 (4)
O7^iii^—Sr1—O7^i^	69.95 (4)	O2^v^—Mn2—O6	97.77 (4)
O5^ii^—Sr1—O7^i^	65.75 (3)	O7—Mn2—O6	60.88 (4)
O4^i^—Sr1—O7^i^	78.31 (3)	O3—P1—O2	111.57 (5)
O6—Sr1—O7^i^	168.81 (3)	O3—P1—O1	107.97 (6)
O3^i^—Sr1—O5^iii^	93.62 (3)	O2—P1—O1	111.08 (6)
O4—Sr1—O5^iii^	68.17 (3)	O3—P1—O4	107.70 (6)
O8^ii^—Sr1—O5^iii^	123.36 (3)	O2—P1—O4	109.65 (6)
O7^iii^—Sr1—O5^iii^	52.59 (3)	O1—P1—O4	108.76 (6)
O5^ii^—Sr1—O5^iii^	143.06 (3)	O5—P2—O6	115.38 (6)
O4^i^—Sr1—O5^iii^	145.93 (3)	O5—P2—O7	113.04 (6)
O6—Sr1—O5^iii^	58.42 (3)	O6—P2—O7	107.71 (6)
O7^i^—Sr1—O5^iii^	113.67 (3)	O5—P2—O8	101.19 (6)
O6—Mn1—O6^iv^	180.0	O6—P2—O8	110.48 (6)
O6—Mn1—O3^v^	92.85 (4)	O7—P2—O8	108.78 (6)
O6^iv^—Mn1—O3^v^	87.15 (4)	O5—P2—Mn2	120.08 (4)
O6—Mn1—O3^i^	87.15 (4)		

Symmetry codes: (i) −*x*+1, *y*−1/2, −*z*+1/2; (ii) −*x*+1, −*y*+1, −*z*+1; (iii) *x*, −*y*+3/2, *z*−1/2; (iv) −*x*+2, −*y*+1, −*z*+1; (v) *x*+1, −*y*+3/2, *z*+1/2; (vi) *x*+1, *y*, *z*; (vii) −*x*+1, *y*+1/2, −*z*+1/2; (viii) *x*, −*y*+3/2, *z*+1/2.

### Hydrogen-bond geometry (Å, º)

**Table:**

*D*—H···*A*	*D*—H	H···*A*	*D*···*A*	*D*—H···*A*
O8—H8···O4	0.82	1.66	2.4828 (14)	177
